# MicroRNA Regulation of Human Protease Genes Essential for Influenza Virus Replication

**DOI:** 10.1371/journal.pone.0037169

**Published:** 2012-05-14

**Authors:** Victoria A. Meliopoulos, Lauren E. Andersen, Paula Brooks, Xiuzhen Yan, Abhijeet Bakre, J. Keegan Coleman, S. Mark Tompkins, Ralph A. Tripp

**Affiliations:** Department of Infectious Diseases, University of Georgia, Athens, Georgia, United States of America; Mayo Clinic, United States of America

## Abstract

Influenza A virus causes seasonal epidemics and periodic pandemics threatening the health of millions of people each year. Vaccination is an effective strategy for reducing morbidity and mortality, and in the absence of drug resistance, the efficacy of chemoprophylaxis is comparable to that of vaccines. However, the rapid emergence of drug resistance has emphasized the need for new drug targets. Knowledge of the host cell components required for influenza replication has been an area targeted for disease intervention. In this study, the human protease genes required for influenza virus replication were determined and validated using RNA interference approaches. The genes validated as critical for influenza virus replication were ADAMTS7, CPE, DPP3, MST1, and PRSS12, and pathway analysis showed these genes were in global host cell pathways governing inflammation (NF-κB), cAMP/calcium signaling (CRE/CREB), and apoptosis. Analyses of host microRNAs predicted to govern expression of these genes showed that eight miRNAs regulated gene expression during virus replication. These findings identify unique host genes and microRNAs important for influenza replication providing potential new targets for disease intervention strategies.

## Introduction

Influenza A viruses generally cause seasonal epidemics however they have the potential to cause pandemics associated with substantial morbidity and mortality [1], [2]. Development of seasonal vaccines is required for influenza virus due to high viral mutation rates that lead to antigenic drift, and also because of periodic antigenic shift which can render vaccines less or ineffective [3]. There are various antiviral drugs that have proven efficacy in the treatment of influenza infections: two M2 ion channel inhibitors (amantadine and rimantadine) and several neuraminidase inhibitors (including zamamivir and oseltamivir) [4], [5], [6], [7], [8], [9]. Early treatment with these antiviral drugs reduces the duration of symptoms and the time to recovery; however, the use of antiviral drugs is complicated by the emergence of drug resistant viruses [5], [6], [10], [11], [12]. In addition, antiviral drug use may come with unwelcome effects that could include an increase in population vulnerability due to lack of seroconversion, as well as driving drug resistance among circulating strains [11]. Thus, it is critical to discover new targets for chemoprophylactics and treatment.

Recent advances in our understanding of RNA interference (RNAi) have provided a means to perform genome-wide screens to determine and validate host cell genes that may be required for influenza virus replication [13], [14]. RNAi is an efficient mechanism for the sequence-specific inhibition of gene expression [15], [16], and is mediated by small interfering RNAs (siRNA) incorporated in the RNA-induced silencing complex (RISC) where the antisense or guide strand of the siRNA can suppress protein expression or direct degradation of messenger RNAs that contain homologous sequences [17], [18], [19]. Synthetic siRNAs can be readily developed to target viral or host genes and have been successfully applied in disease intervention approaches. For example, siRNA targeting respiratory syncytial virus has shown efficacy for silencing virus replication [20], [21], [22], [23], [24], [25], a feature that has led to RNAi-based clinical trials as a new therapeutic option [23]. In addition, there are promising results from targeting host genes, such as the use of siRNA silencing for the treatment of age-related macular degeneration [26], and in the case of influenza, inhibiting the host gene CAMK2B prevented vRNA transcription in vitro [27], and shRNA inhibition of trypsin also inhibited replication and apoptosis [28]. Recently, several studies employed genome-wide RNAi screens to identify host genes required for influenza virus infection and replication [27], [29], [30], [31], [32], and genes have also been identified by random homozygous gene perturbation [33] and by a proteomic screen [34]. Although there were few common genes detected among the studies, meta-analysis revealed that influenza virus was co-opting many of the same host cell pathways [27], [29], [30], [31], [32], [35]. Thus, the inability to find the same genes among the studies is not unexpected given that multiple genes may be affected in the same host cell pathway, that the tempo of gene expression may vary among the cell lines studied, and that differences can be attributed to variations in methodologies, viruses, and cell lines used among the studies [27], [29], [30], [35].

**Figure 1:**
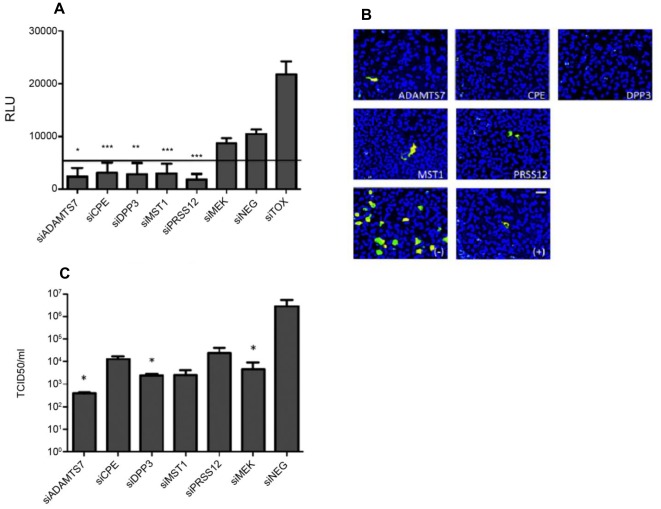
RNAi of 5 host protease genes down-regulated influenza virus replication. A: A549 cells were reverse transfected with 50 nM of siRNA (SMARTpool) specific for the indicated genes (ADAMTS7, CPE, DPP3, MST1, PRSS12). After 48 hours, cytotoxicity was determined by adenylate kinase (AK) release. Cells treated with the siTOX control were considered 100% cytotoxic and all values were normalized to siTOX. RLU = relative luciferase units. * p<0.05 vs siNEG, ** p<0.01 vs siNEG, ***p<0.005 vs siNEG; siTOX vs all samples: p<0.001 (not shown). Line shows 20% of siTOX control. B: A549 cells were reverse transfected with 50 nM of siRNA (SMARTpool) specific for the indicated genes (ADAMTS7, CPE, DPP3, MST1, PRSS12). After 48 hours, cells were infected with A/WSN/33 at an MOI of 0.001. 48 hours post-infection, cells were fixed in 4% formaldehyde and stained with an anti-NP (green) monoclonal antibody followed by counterstain with DAPI (blue.) Positive (+) control: siMEK, negative (−) control: siNEG. Magnification is 20× (bar is 100 microns). C: Cells were transfected with 100 nM of a novel siRNA targeting a different seed site from the SMARTpool used in the primary screen and infected as in B. After 48 hours of infection, cellular supernatant was tested for infectious virus production by a modified TCID_50_. Data is expressed as TCID_50_/ml. Data is representative of two independent experiments. (*p<0.05 vs siNEG).

Of the host genes known to affect influenza virus, the proteases are important for infection and replication. Proteases may affect virus infection and replication in several ways including viral entry and hemagglutinin (HA) processing [36], [37], [38], [39], degradation of viral components for MHC presentation [40], cap-snatching [41], induction of apoptosis [42], and by increasing vascular permeability aiding in the development of systemic infection in cases of severe infection [43]. However, it remains unclear which protease genes are essential in the biology of influenza virus replication.

**Table 1:** Human protease gene hits.

Gene	Name	Accession number	siRNA sequences	Function
ADAMTS7	ADAM metallopeptidase with thrombospondin type 1, motif 7	NM_014272	SMARTpool * :GCGAGGACCCGGAGAAGUAGAACGUGGGCUGUGACUUCCAACGAGGACUACUUCAUUGCAGAUACUGUGUGGGUGANovel siRNA for Validation # :CCAAGGACAUUAUCGACUU	Role in inflammation of extracellular matrix [56], [77], [78], [79]
CPE	Carboxypeptidase E	NM_001873	SMARTpool * :GAAAGAAGGUGGUCCAAAUGCUUAUACCUGGAAACUAUGGAAUAGACCACGAUGUUAGGAUGCAAGACUUCAAUUANovel siRNA for Validation # :GAUGAGACGCGGAGUGGUA	Role in local opioid network in the lung [57], [80], [81], [82]
DPP3	Dipeptidyl-peptidase 3	NM_005700	SMARTpool * :ACACGGUGCUGCUGCGUAAGAGGGAAUCACCACCUAUUGCAGCAAGAUCCGGUCUGUGCUCAGACGUGCAGCUUCUNovel siRNA for Validation # :GAUCCUUCUCUGAGCGUUU	Role in inflammation and apoptosis [83], [84], [85]
MST1	Macrophage stimulating 1 (hepatocyte growth factor-like)	NM_020998	SMARTpool * :GUACGGACCUGCAUCAUGAGGAAUGGCCUGGAAGAGAAGCUGUGACCUCUUCCAGAACGACAACUAUUGCCGGAAUNovel siRNA for Validation # :GACCAAAGGUACGGGUAAU	Role in inflammation and response to tissue injury [55]
PRSS12	Neurotrypsin, motopsin	NM_003619	SMARTpool * :GGACUGAGCUGAAUACAUAGAUGAUGGAUGGACUGAUAGAGCAUAACUGUGGCCAUAGGACGAGCCUAUUCAAGAANovel siRNA for Validation # :GAGCAAGAACCAUGGCUUA	Unknown

* : Refers to the pool of four siRNAs used in the primary screen.

# : Refers to the individual siRNA used for the validation step.

In addition to host gene involvement during viral infection, the tempo of host gene expression may be altered by a variety of factors, such as by microRNAs (miRNA). Host miRNAs are similar to siRNAs in their silencing mechanism, but miRNAs are generated in the nucleus from short hairpin precursors and must be processed and exported before entering the RISC pathway [44]. miRNAs can be generated by the virus [45], [46] or the host and can silence genes by similar mechanisms to siRNAs; however, unlike siRNA silencing, miRNA sequences do not need to be homologous to the target mRNA [44]. Little is known about the role of miRNAs during virus infection, however host-derived miRNAs have been shown to negatively affect influenza replication [47] and miRNAs have even been used as therapeutics [48], [49]. We considered that host miRNAs might also have a role in host gene modulation during influenza infection.

**Figure 2:**
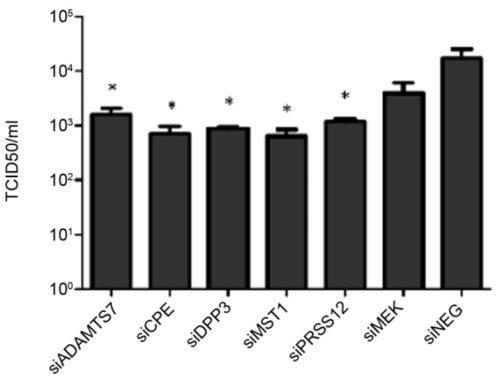
RNAi of individual host protease genes down-regulates replication of a clinical influenza isolate. A549 cells were reverse transfected with 100 nM of the novel siRNA targeting siADAMTS7, siCPE, siDPP3, siMST1, and siPRSS12. After 48 hours, cells were infected with A/New Caledonia/20/99 at an MOI of 0.1 in the presence of 1 ug/ml TPCK-trypsin. After 48 hours of infection, cellular supernatant was tested for infectious virus production by a modified TCID_50_. Data is expressed as TCID_50_/ml. Data is representative of two independent experiments. (*p<0.05 vs. siNEG).

**Figure 3:**
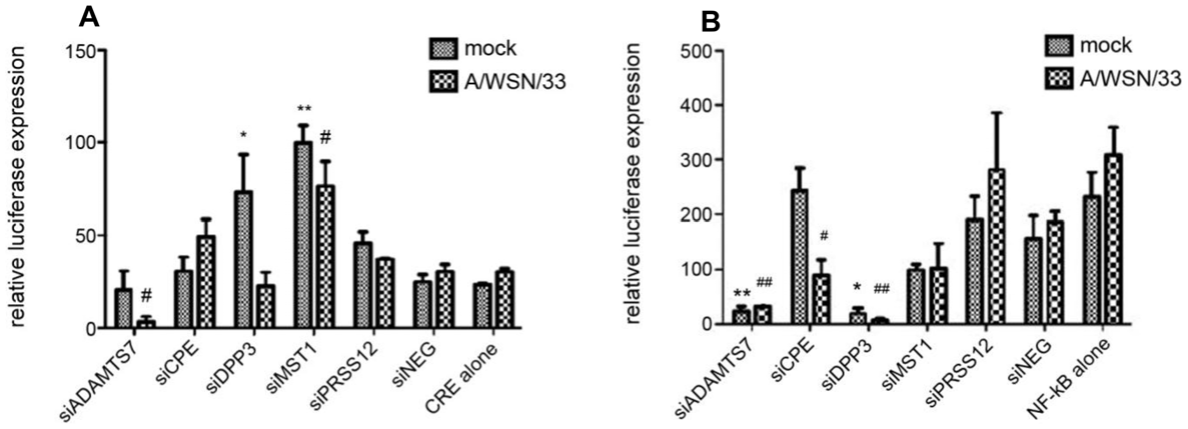
Analysis of host gene involvement in major cellular pathways. A: A549 cells were reverse cotransfected with CRE/CREB reporter plasmid or the appropriate control plasmid and 100 nM of the novel siRNA. After 24 h incubation, the transfection media was replaced with culture media. Cells were mock infected or infected with A/WSN/33 at an MOI of 0.001 the following day. After 24 h, culture supernatant was analyzed for luciferase expression. Luciferase units were normalized to Renilla expression. * p<0.05, ** p<0.01 compared to siNEG control (mock), # p<0.05, ## p<0.01 compared to siNEG control (A/WSN/33) B: A549 cells were reversed cotransfected with NF-κB reporter plasmid or the appropriate control plasmid and 100 nM of the novel siRNA. After 24 h incubation, the transfection media was replaced with culture media. Cells were mock infected or infected with A/WSN/33 at an MOI of 0.001 the following day. After 24 h, culture supernatant was analyzed for luciferase expression. Luciferase units were normalized to Renilla expression. * p<0.05, ** p<0.01 compared to siNEG control (mock), # p<0.05, ## p<0.01 compared to siNEG control (A/WSN/33). Data is representative of three independent experiments.

To comprehensively determine the host proteases required by influenza virus for infection and replication, a siRNA screen of all known 481 protease genes was performed in human lung epithelial (A549) cells using the influenza virus, A/WSN/33. Twenty-four primary gene hits were found in the siRNA screen of which five (ADAMTS7, CPE, DPP3, MST1, and PRSS12) were validated as critical for replication. Pathway analysis revealed that these five genes were linked to cAMP responsive binding element (CREB), NF-κB, and apoptosis pathways, thus we examined and verified host gene regulation of these pathways. As the tempo of host gene expression is governed by miRNAs which have important roles in regulating host genes during viral infection and replication [45], [50], [51], an additional analysis was performed to identify miRNAs that might potentially regulate the five validated host genes required for influenza virus replication. A library of miRNA antagonists was used to confirm miRNA regulation, and several miRNAs were identified to affect virus replication and host gene regulation, notably miR-1254, miR-106B, miR-106B*, miR-124-a, and miR-124*. This finding shows varied miRNA expression patterns associated with the regulation of various genes involved in global host cell pathways governing inflammation (NF-κB), cAMP/calcium signaling (CRE/CREB), and apoptosis, and adds to our understanding of how miRNAs may govern host gene regulatory networks involved in the response to influenza virus replication.

**Figure 4:**
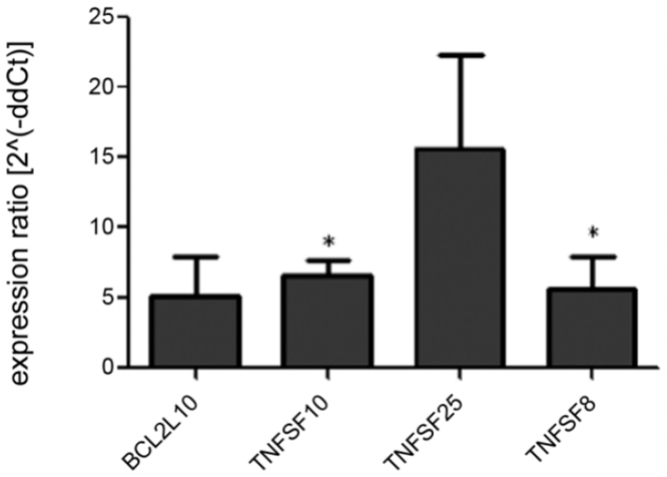
RNAi of DPP3 (siDPP3) inhibits influenza replication by modulation of apoptotic genes. A549 cells were reverse transfected with 50 nM of siDPP3 or siNEG. After 48 hours, cells were infected with A/WSN/33 at an MOI of 0.001. After 18 hours of infection, cellular RNA was isolated and apoptosis gene expression profiles were determined by array. Gene expression was normalized to GAPDH levels. Silencing DPP3 resulted in upregulated levels of the pro-apoptotic genes BCL2L10, TNFSF10, TNFSF25 and TNFSF8. Data is representative of three independent experiments. * p<0.05.

## Results

### Human protease genes required for influenza replication

A primary RNAi screen of 481 host protease genes in a human type II respiratory epithelial cell line (A549) identified 24 genes important for A/WSN/33 influenza virus replication (Figure S1). The gene hits identified were ≥1.5σ and ≤−1.5σ from the mean Z-score. Endpoint validation of the gene hits in the primary screen included influenza nucleoprotein (NP) cell localization determined by immunohistochemistry, determining the level of infectious virus by TCID_50_ assay, as well as influenza matrix (M) gene copy number determined by qPCR (data not shown). For validation of primary gene hits, a novel siRNA targeting the same gene but at a different seed site (Table 1) was required to produce the same phenotype as observed in the screen. Protein levels of the protease genes were assessed to ensure that RNAi silencing was decreasing target gene expression (Figure S2). After validation, five genes were identified that decreased virus replication when silenced: ADAMTS7, CPE, DPP3, MST1, and PRSS12 (Table 1). Silencing of these protease genes did not cause cytotoxicity in A549 cells compared to the cytotoxicity (siTOX) control. siMEK (an siRNA targeting MAP2K) and siNEG (a negative, non-targeting siRNA control) both displayed low level cytotoxicity possibly due to the importance of MEK in cellular signaling, and the induction of an inflammatory response to siNEG due to off-target effects (Figure 1A). Silencing of the five validated genes resulted in considerably decreased influenza NP staining compared to the negative (−) control, and was consistent with low NP staining in cells treated with the positive (+) siRNA control, siMEK (Figure 1B). The level of infectious virus from A549 cells treated with 100 nM of a single siRNA targeting each of the validated genes was greatly (siCPE, siMST1 and siPRSS12) and significantly (p<0.05; siADAMTS7 and siDPP3) reduced (Figure 1C). Influenza virus M gene levels reflected the low level of infectious virus and NP staining observed for A549 cells treated with siRNAs targeting the five validated genes, as well (data not shown).

**Figure 5:**
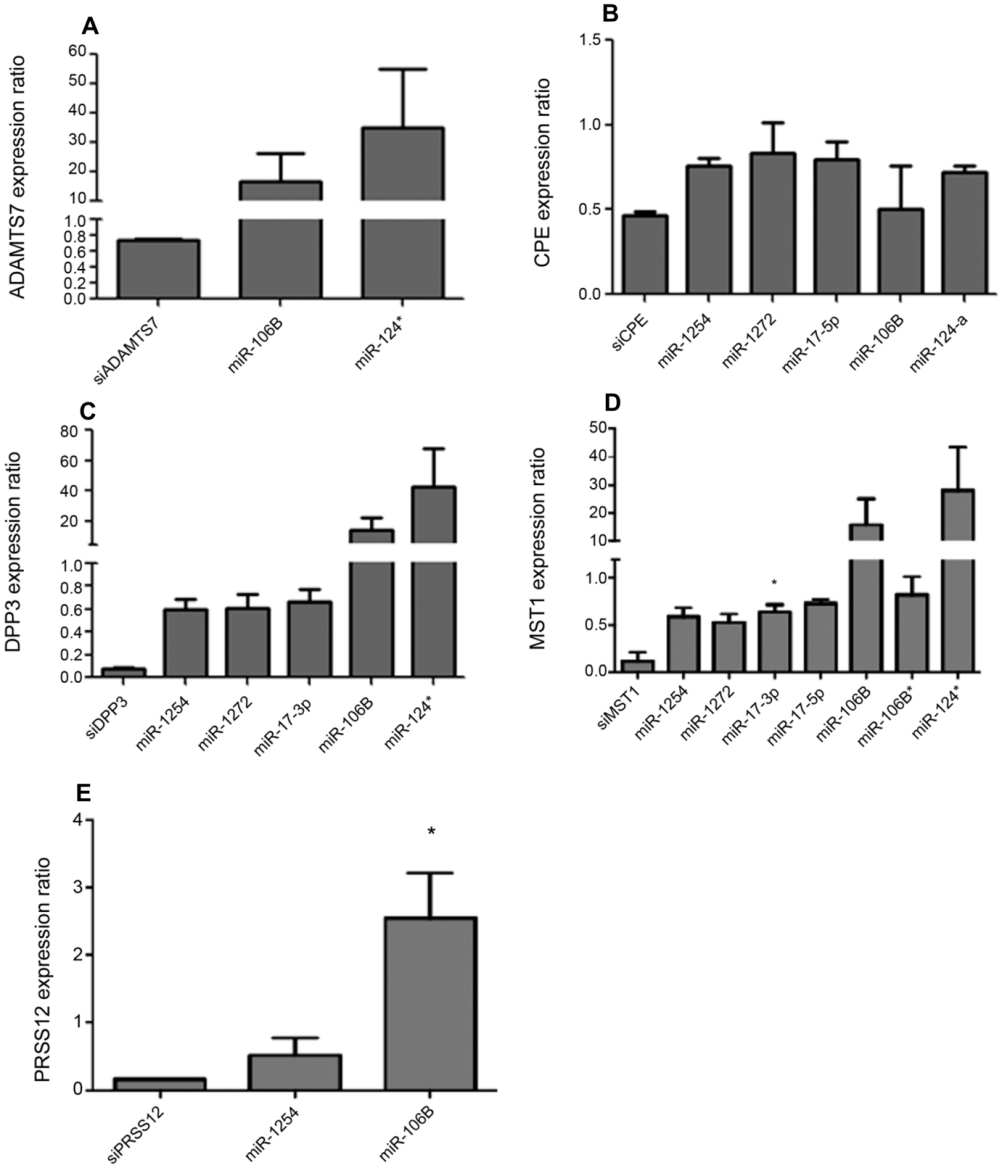
Effect of miRNA inhibition on host protease gene expression 24 h post-miRNA inhibitor treatment. Host cell miRNAs of interest were evaluated for their effect on host gene hits by qPCR. A549 cells were treated with the appropriate miRNA inhibitor (25 nM) for 24 hours. Cellular RNA was isolated 24 hpi and evaluated by qPCR for host gene expression using a SYBRgreen assay with gene-specific primers. Gene expression was compared to cells transfected with siNEG (for siRNA) or NEG (non-targeting miRNA inhibitor) at the equivalent concentration. Data is normalized to GAPDH expression. miRNAs indicated on the x-axis refer to inhibition of those miRNAs. A: ADAMTS7 expression levels, B: CPE, C: DPP3, D: MST1, E: PRSS12. Data is representative of two independent experiments. (*p<0.05 versus siRNA treatment.).

### Host genes validated with a clinical influenza virus isolate

Since A/WSN/33 was used in the primary and secondary screens because of its ability to grow in the absence of trypsin, a feature facilitating high throughput screening, cross-validation of ADAMTS7, CPE, DPP3, MST1, and PRSS12 genes was performed in A549 cells with A/New Caledonia/20/99. Using the same siRNAs previously used to validate the genes (Figure 1C), A549 cells treated with 100 nM of the siRNAs significantly (p<0.05) reduced A/New Caledonia/20/99 virus replication (Figure 2). The differences in viral titer were at least 1.5 logs lower compared to siNEG-treated cells for all five genes examined. These results show that ADAMTS7, CPE, DPP3, MST1, and PRSS12 contribute to influenza A virus replication, and provide promising disease intervention targets.

**Figure 6:**
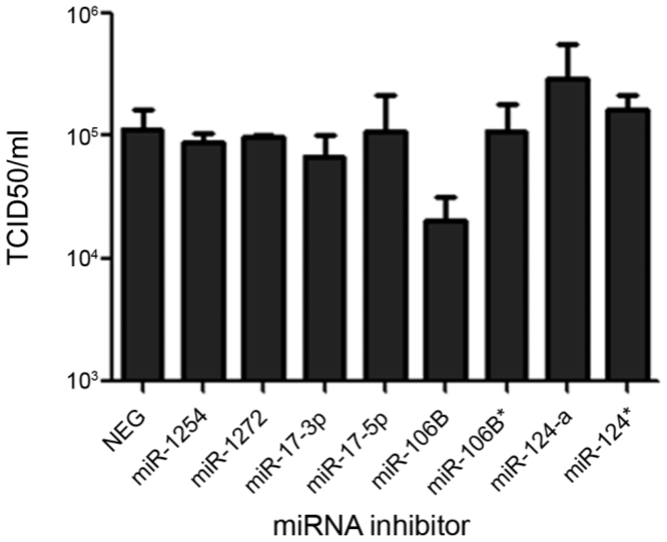
Effect of miRNA inhibition on influenza replication. A549 cells were treated with the appropriate miRNA inhibitor (25 nM) for 48 hours, followed by infection with A/WSN/33 (MOI = 0.001). Cellular supernatant was tested for infectious virus production by a modified TCID_50_ 48 hpi. Data is expressed as TCID_50_/ml and is representative of two independent experiments.

### Cell pathways associated with the validated host genes affect influenza replication

Expanding from the primary screen toward defining host cell pathways involved in influenza virus replication requires assessment of the signaling pathways, molecular networks, and biological processes that are linked to the five validated host genes. Dynamic pathway analysis identified three major pathways for the five validated genes, i.e. NF-κB pathway, CRE/CREB signaling pathway, and cellular apoptosis (Figure S3). To verify the host genes in these pathways, A549 cells were transfected with a luciferase assay reporter plasmid governed by the promoter of interest linked to a firefly luciferase gene. The level of pathway activation was determined by luciferase expression, which was normalized to a control plasmid expressing Renilla luciferase to account for transfection efficiency. Pathway analysis suggested that MST1 and PRSS12 genes participate in the CRE/CREB signaling pathway. RNAi of MST1 by siRNA targeting (siMST1) led to a significant (p<0.05) increase in CRE/CREB signaling compared to the siNEG and CRE controls in the presence or absence of influenza virus infection (Figure 3A). RNAi of PRSS12 by siPRSS12 did not affect CRE/CREB activation relative to controls. Interestingly, RNAi of DPP3 using siDPP3 significantly (p<0.05) increased CRE/CREB activation under mock-infected conditions despite DPP3 not being implicated in the CRE/CREB pathway, and silencing of ADAMTS7 significantly (p<0.05) abrogated CRE/CREB activation during infection. Pathway analysis also indicated that ADAMTS7, CPE, and MST1 genes were potentially involved in the NF-κB activation pathway (Figure S3). siADAMTS7 significantly (p<0.01) abrogated NF-κB activation regardless of infection or mock-treatment (Figure 3B). Interestingly, a similar finding was observed for DPP3, a gene not previously linked to the NF-κB signaling pathway by our analysis. siCPE did not affect NF-κB activation under mock conditions, but during infection NF-κB activation levels were significantly (p<0.05) downregulated. siMST1 also had a negative effect on NF-κB activation regardless of infection. Elevated NF-κB levels were also seen in controls receiving the NF-κB plasmid alone but this was not significant compared of siNEG controls.

Dynamic pathway analysis predicted DPP3 to be involved in apoptotic pathways (Figure S3), and as a luciferase/Renilla pathway reporter system was unavailable, a human apoptosis array was performed to determine the level of mRNA expression of various pro- and anti-apoptotic host genes in relation to DPP3 expression (Figure 4). After confirming RNAi silencing (>95%) of DPP3 by PCR (data not shown), gene expression was compared in cellular RNA extracted from infected A549 cells treated with either siDPP3 or siNEG (50 nM). While most genes tested did not vary in expression levels relative to siNEG-treated cells, four pro-apoptotic genes were found to be substantially increased when DPP3 was silenced: BCL2L10, TNFSF10, TNFRSF25, and TNFSF8. Of these four genes, TNFSF10 was significantly (p<0.05) increased 6-fold, and TNFSF8 was significantly (p<0.05) increased 5-fold compared to siNEG control treated cells. TNFRSF25 was substantially increased as well. These findings show that inhibition of DPP3 expression initiates higher expression of pro-apoptotic factors, features that may negatively impact influenza virus replication and associated modulation of cellular apoptosis. Silencing of the other four target protease genes did not significantly affect levels of pro- or anti-apoptotic genes compared to cells treated with siNEG as predicted by the Ingenuity pathway analysis, although TNFSF10 was substantially increased when cells were treated with siADAMTS7 (Table S1).

### miRNAs govern host genes required for influenza replication

Pathway analysis of the five validated host genes revealed potential miRNA interaction (Figure S4) with eight miRNAs (miR-1254, miR-1272, miR-17-5p, miR-17-3p, miR-106B, miR-106B*, miR-124-a, and miR-124*). To determine the role of these miRNAs in regulating ADAMTS7, CPE, DPP3, MST1, and PRSS12, A549 cells were treated with miRNA hairpin inhibitors as previously described [52], or treated with a negative control from *C. elegans* that shares no homology with known human miRNA sequences, and gene expression levels determined by qPCR. As gene modulation by miRNAs is usually subtle and multi-targeted, the effect of the miRNA inhibitors on host gene mRNA levels was determined 24 hours post-treatment. All 8 miRNA inhibitors were tested for their effect on each of the 5 validated genes; however, only those inhibitors that affected gene expression are shown (Figure 5). At 24 h post-treatment, ADAMTS7 gene expression levels increased 20-fold when miR-106B was inhibited, and 40-fold when miR-124* was inhibited (Figure 5A). Treatment with siRNA targeting the gene examined was performed as a control. CPE expression was slightly decreased by miRNA inhibitors, as there was no response above 1.0 (Figure 5B). In contrast, inhibition of miR-106B and miR-124* resulted in a >20-fold and >40-fold increase of DPP3 gene expression, respectively (Figure 5C), while miR-1254, miR-1272, and miR-17-3p inhibition caused a decrease of DPP3 expression. MST1 expression was increased to similar levels as DPP3 with the same miRNA inhibitors, while the other miRNA inhibitors resulted in a slight (but significant for miR-17-3p) decrease in expression (Figure 5D). Finally, PRSS12 expression levels were significantly (p<0.05) increased in response to miR-106B inhibition, but a slight decrease was detected by inhibition of miR-1254 (Figure 5E). These results show the same miRNAs can regulate different genes both subtly and robustly.

Given the evidence that miR-1254, miR-1272, miR-17-5p, miR-17-3p, miR-106B, miR-106B*, miR-124-a, and miR-124* are involved in governing aspects of ADAMTS7, CPE, DPP3, MST1, and PRSS12 gene expression (Figure 5), the role of these miRNAs in the regulation of influenza virus replication was determined (Figure 6). A549 cells were treated with individual miRNA inhibitors and the cells infected with A/WSN/33 to determine the effect on virus replication (Figure 6). Of the 8 miRNA inhibitors tested, inhibition of miR-106B was associated with a decrease in influenza virus replication, while inhibition of miR-124 resulted in an increase in virus replication with respect to the negative control. Inhibition of the other eight miRNAs had more subtle effects with slight increases or decreases of influenza virus replication. The decrease of virus replication associated with inhibition of miR-106B is likely associated with a decrease of CPE gene expression as RNAi silencing of CPE was associated with low levels of virus replication (Figures 1 and 2). The level of virus replication was confirmed by qPCR M gene levels and was consistent with the findings observed in Figure 5 (data not shown). The results show that some of the miRNAs modulate host genes critical for influenza virus replication, thus it likely that the tempo of host gene expression is differentially regulated in response to influenza virus infection. Further, the results provide evidence that targeting miRNAs may offer an alternative disease intervention approach to control influenza virus replication.

## Discussion

Some human proteases are known to have a direct function in the replication of influenza virus [38], [39], but the role of other proteases in the biology of virus replication and host cell pathways they affect are not fully elucidated. To better understand protease gene requirements for influenza virus replication, and discover novel disease intervention targets, we conducted an RNAi screen of 481 genes comprising the human protease genome and validated five genes, ADAMTS7, CPE, DPP3, MST1, and PRSS12 that are required for influenza replication. The primary and secondary RNAi screens were performed with A/WSN/33 influenza virus because of its ability to replicate in the absence of trypsin in a type II respiratory epithelial (A549) cell line. All five genes showed reduced A/WSN/33 titers and some showed a substantial decrease in NP staining to the level where NP was not detected using a NP-specific monoclonal antibody. This may have been due to an inability of the virus to reenter the host cells for subsequent infection as a consequence of protease gene silencing. To confirm the findings for WSN/33 infection, the five validated genes were assessed against a clinical influenza virus strain, i.e. A/New Caledonia/20/99. RNAi silencing of all five genes resulted in decreased levels of A/New Caledonia/20/99 replication. Although previous RNAi host factor screens for influenza virus replication did not identify any of the five protease genes in this study, the same host cell pathways were identified which included the genes validated in this study [27], [29], [30], [31], [32]. Specifically, our pathway analysis for this study showed that ADAMTS7, CPE, DPP3, MST1, and PRSS12 were involved in three previously identified host cell pathways, specifically CRE/CREB signaling, NF-κB activation, and apoptosis. It is likely that different pathway interaction is co-opted by the virus at different stages in the virus replication process, but further study is needed to determine the exact process. An additional caveat may be the use of an immortalized cell line to identify gene hits. Clonal cell lines can have misregulated gene expression relative to primary cells, particularly for genes involved in cell growth and differentiation [53]. Use of a primary human cell line to perform this study, e.g. normal human bronchial epithelial (NHBE) cells may be more desirable; however, primary cells often have reduced transfection efficacy and issues with maintenance of a uniform stage of differentiation that makes it difficult to perform high-throughput screening [35]. Therefore, it is possible that some genes discovered may not translate to a primary cell line, so further evaluation is needed to confirm the results of this study.

CRE/CREB signaling is an important cellular process that serves a variety of functions. Most notably with respect to influenza infection, CRE/CREB signaling has been shown to activate protein kinase A (PKA) and thus have a role in protein synthesis [54]. In this study, MST1 and PRSS12 were implicated in CRE/CREB signaling; however, in response to influenza infection, CRE signaling levels in cells treated with siPRSS12 were unchanged compared to both pathway reporter and siNEG controls. However, RNAi of the MST1 gene resulted in significantly higher CRE signaling levels regardless of infection. These findings may suggest that MST1 is involved in some aspect of CRE signaling or the cAMP response, and without MST1, the calcium response element is not activated.

ADAMTS7, CPE, and MST1 have been shown to have a role in tissue injury and inflammation [55], [56], [57], and pathway analysis performed in this study identifying their linkage to NF-κB pathway activation is consistent with these earlier findings. However, there was no significant effect of these genes on the NF-κB pathway relative to controls. However, A549 cells treated with siRNAs targeting ADAMTS7 and DPP3, a gene not predicted to be involved in the NF-κB pathway, resulted in complete abrogation of NF-κB regardless of influenza infection. This finding suggests that NF-κB signaling may involve ADAMTS7 and DPP3. As ADAMTS7 plays a role in maintenance of the extracellular matrix [56] and DPP3 is a metallopeptidase, it is possible that silencing these genes is decreasing the inflammatory response because less damage is being done to basement tissue. ADAMTS7 has also been shown to be upregulated by TNF-α and IL-1β secretion [56], both of which are activated upon influenza infection [43].

DPP3 is involved in apoptosis modulation and is over-expressed in cancerous cells [58]. RNAi of the DPP3 gene was performed to evaluate the effect on apoptotic gene expression. The results showed induction of BCL2L10, TNFSF10, TNFRSF25 and TNFSF8 pro-apoptotic genes. BCL2L10 is a pro-apoptotic gene that interacts with caspase 9 to signal apoptosis [59], and TNFSF8 and TNFRSF25 are involved in the pro-inflammatory response through NF-κB signaling [60], [61]. TNFSF10, also known as TRAIL, has been shown to be involved in H5N1 virus-induced apoptosis in human monocyte-derived macrophages [62]. Since DPP3 silencing resulted in a sharp decrease of influenza replication as quantified by TCID_50_ and NP staining, the findings suggest that reducing DPP3 gene expression causes influenza virus infected cells to initiate apoptosis without interference by the known anti-apoptosis activities of the influenza NS1 protein [3]. It is possible that without the NS1-mediated apoptosis delay, the virus is not able to replicate and bud before the host cell is eliminated. Furthermore, as NF-κB appears to have a role in apoptosis modulation, the effect of DPP3 silencing on NF-κB activation observed in this study is predictable and consistent with earlier findings [63].

Host gene expression is governed by miRNAs [44], [64], thus it is important to understand potential miRNA regulation of host genes validated as required for influenza virus replication. The results from the miRNA hairpin inhibitor studies showed that targeting the predicted miRNAs had a considerable and significant outcome on most host gene expression. For example, inhibition of miR-106B and miR-124* resulted in 20-fold and 40-fold increased ADAMTS7 gene expression levels, respectively, while CPE gene expression was slightly reduced by miRNA inhibitors. As expected, some miRNA inhibitors had differential effects on host genes required for influenza virus replication. For example, inhibition of miR-106B had little effect on CPE gene expression, but dramatically increased DPP3 gene expression. These findings show that expression of host genes required for influenza virus replication can be regulated by multiple miRNAs, and suggests that targeting miRNAs to regulate host gene expression may be a strategy to regulate influenza virus replication.

As predicted from the host gene expression studies, miRNA inhibitors also affected influenza virus replication. For example, inhibition of miR-106B, a miRNA known to cause cell cycle arrest when inhibited in a laryngeal cancer model [65], resulted in substantially decreased virus replication. Human papillomavirus oncoproteins E6 and E7 have also been reported to dysregulate miR-106B [66]. Inhibition of miR-124-a resulted in an increase of influenza virus replication relative to the negative control. Although miR-124-a was not found to regulate any of the 5 host protease genes from our screen, it is reported to have a putative target in both swine influenza virus and the 2009 pandemic H1N1 strain [67]. Inhibition of other miRNAs also subtly modulated infectious virus levels, but not to a level significantly different from the NEG control. In this study, inhibition of miR-106B increased ADAMTS7, DPP3, MST1 and PRSSS12 gene expression, but subtly reduced CPE gene expression, an effect that resulted in reduced influenza virus replication, implying the effect of miR-106B inhibition on CPE may be dominant. Additionally, inhibition of miR-1254 resulted in slightly decreased CPE, DPP3, MST1 and PRSS12 gene expression levels, and this translated to slightly decreased influenza virus replication. Since none of the 5 validated genes were increased by inhibition of miR-124-a, it is likely miR-124-a affects influenza replication through other genes yet identified. These findings indicate that while miRNAs do modulate host gene expression, they likely modulate influenza replication indirectly and perhaps at different points in the replication pathway. Further study is needed to validate miRNA involvement and mechanism of virus inhibition or assistance. Taken together, these findings suggest novel targets and potentially new therapies for regulating influenza replication and the pathways co-opted during infection.

## Materials and Methods

### Cells and virus stocks

A549 cells (ATCC CCL-185) were cultured in Dulbecco's modified Eagle's medium (DMEM) (HyClone, Logan, UT) containing 5% heat-inactivated FBS (HyClone, Logan, UT). Cells were frozen in 10% DMSO and 90% FBS to create one stock of a single cell passage that was used for the entire screen. A stock of MDCK cells (ATCC CCL-34) was also propagated and stored using the same conditions. A/WSN/33 (H1N1) influenza virus was used for the primary siRNA screen as this virus has the ability to replicate without the need for exogenous trypsin [68]. For the validation studies, A/New Caledonia/20/99 (H1N1) influenza virus was also used. All viruses were propagated in 9-day-old embryonated chicken eggs as previously described [69]. Viruses were titrated in MDCK cells and titers calculated by the method developed by Reed and Muench [70].

### Protease library screen

A primary screen using four pooled siRNAs to target each gene of the 481 genes in the human protease library (SMARTpool; Dharmacon ThermoFisher, Lafayette, CO) was performed using A/WSN/33 influenza virus and type II human alveolar pneumocytes (A549 cells) similar to a method previously described [27]. siRNAs were resuspended in Dharamcon siRNA buffer to a concentration of 1 uM and stored at −80°C until use. The screen was conducted in two steps, a primary screen targeting all 481 human protease genes, followed by a smaller-scale validation screen of the primary hits to confirm the genes identified were essential for influenza virus replication. In all studies, a siRNA targeting the MEK gene (siMEK), a well-characterized human kinase gene important for influenza replication [4], [71], was used to control for the transfection efficiency and host gene silencing. A non-targeting siRNA control (siNEG) was also used in all assays. The siRNA SMARTpool library constituents, siMEK, and siNEG transfected A549 cells at >85% efficiency, and a few (<2%) siRNA pools targeting HP genes induced cytotoxicity as determined by adenylate kinase (AK) release in the cell culture supernatant [72], [73] and by visual inspection of cell morphology.

A549 cells were reverse transfected with the siRNA 48 hours before infection with influenza A/WSN/33 (MOI = 0.001). The amount of infectious virus was measured 48 hpi by titration of A549 cell supernatant on MDCK cells, and the results normalized to siNEG-treated cells. All assays were run in duplicate and the entire screen assay was repeated twice. Both plate-based controls and assay-wide controls, e.g. siMEK, siNEG, and siTOX, were included on each individual assay plate. The results were normalized to the plate median for the SMARTpools. After excluding cytotoxic siRNAs based on detection of AK, primary screen data was subject to analysis. The primary screen, performed as two independent studies, was analyzed using a scaling methodology that sets the non-targeting control siRNA (siNEG) at an arbitrary value of 1.0, and the negative control siTOX at zero. siRNAs targeting host genes were assigned a score based on the distribution of these values. Wells in the primary screen with a percent of differentiation ≥|1.5| standard deviations from the plate mean in both duplicate assays were considered primary hits. Of the 481 HP genes targeted, twenty-four genes were identified as “primary hits”. Hits that caused greater than 20% cytotoxicity in A549 cells were excluded (Figure 1A). Z-scores were computed based on this data for each gene hit and those ≥1.5σ and ≤−1.5σ were considered for validation.

### Reverse transfection

Lyophilized siRNAs in 96-well plates were diluted with HBSS (HyClone, Logan, UT) and allowed to incubate for 5 minutes. Dharmafect-1 transfection reagent (Lafayette, CO) and HBSS were added such that each well received 0.004 ml of transfection reagent and 0.096 ml of HBSS. The siRNA/transfection reagent mix was allowed to incubate for 20 minutes at room temperature after which 0.08 ml of 1.5×10^4^ A549 cells suspended in DMEM/5% FBS was added to each well, and the plate incubated for 48 hours at 37°C in 5% CO_2_. The final concentration of siRNA for all primary screen transfections was 50 nM.

### Cytotoxicity and virus infection

To determine if siRNA gene silencing was cytotoxic, the cell supernatants from siRNA transfected A549 cells were analyzed for adenylate kinase (AK) using a Toxilight kit (Lonza, Rockland, ME). Results were normalized to a siTOX control, i.e. a siRNA control (Dharmacon) causing complete cell death by 48 hours. siRNA transfected cells with luminescence greater than or equal to 20% of the siTOX control were not considered for further evaluation. A549 cells were subsequently infected with A/WSN/33 at an MOI of 0.001 pfu/cell. Cells were incubated for 48 hours at 37°C/5% CO_2_.

### Endpoint assays

Virus titers in siRNA-treated A549 cells infected with A/WSN/33 were determined by modified TCID_50_ or hemagglutination assay (HA). Briefly, virus infected A549 cell culture supernatants were serially diluted ten-fold, and added to MDCK cells. The MDCK cell plates were incubated for 72 h, followed by an HA using 0.5% chicken red blood cells as previously described [74]. To further identify gene hits the cells were screened for nucleoprotein (NP) by IHC staining and evaluated using high content analysis. For NP staining, the cells were fixed with 3.7% formaldehyde and stained with anti-NP monoclonal antibody (5 ug/ml; H16-L10-4R5) and the antibody staining detected using Alexa Fluor 488 labeled goat anti-mouse IgG (1 ug/ml; Invitrogen, Carlsbad, CA). Cells were counterstained with DAPI (2 ug/ml) (Invitrogen, Carlsbad, CA) and visualized by immunofluorescent microscopy (20×, EVOS digital inverted fluorescent microscope, Advanced Microscopy Group, Bothell, WA). As an additional hit identification endpoint, real-time qRT-PCR analysis was performed to quantify influenza M gene copy number in the cells as previously described [75]. Briefly, RNA was purified using the RNeasy kit (Qiagen, Valencia, CA), and cDNA was synthesized using a SuperScript First Strand cDNA synthesis kit (Invitrogen, Carlsbad, CA) and appropriate primer/probe set [75] to detect the M gene. PCR was performed using the amplification cycle: 10 minutes at 95°C followed by 40 cycles of 95°C for 30 seconds, 60°C for 1 minute, and 72°C for 30 seconds. M gene copies were normalized to the siNEG control.

### Validation of gene hits

Individual novel siRNAs (Dharmacon) were used to target a different seed site on the same gene for gene hits identified during the primary screen (Table 1). Gene silencing was confirmed by qPCR using SybrGreen (Qiagen, Valencia, CA) to detect the dsDNA product allowing for quantification of gene silencing relative to cells treated with the siNEG control. Primers targeting each individual hit were compared to control GAPDH levels. For phenotype validation, A549 cells were transfected with 100 nM siRNA, incubated 48 h at 37°C in 5% CO_2_, infected with A/WSN/33 (MOI = 0.001), and the amount of infectious virus was measured 48 hpi by TCID_50_ and NP staining assays to validate the screen phenotype previously observed. From the primary gene hits, five genes essential for influenza virus replication were validated using the novel siRNAs. The five genes were also tested using A/New Caledonia/20/99 at an MOI of 0.001 as described above; however infection was done in the presence of TPCK-trypsin (1 ug/ml).

### Western blot

To determine protein levels in A549 cells treated with siRNA specific for the target protease genes, A549 cells were untreated, treated with 50 nM of the siNEG control, or treated with 50 nM of the appropriate siRNA. After 72 hours, cells were lysed in 1% TritonX-100 and proteins from total cell lysate were separated on a 4–20% Tris-HCl precast SDS PAGE gel (BioRad, Hercules, CA) and transferred to a PVDF membrane. Membranes were blocked in 5% BSA/TBS- 0.05% tween and incubated with 1 ug/ml rabbit polyclonal primary antibodies to ADAMTS7, CPE, DPP3, MST1, or PRSS12 (Abcam, Cambridge, MA). A goat anti-rabbit alkaline phosphatase-conjugated secondary antibody (Invitrogen) was used to visualize the proteins.

### Host cell pathway analysis

To survey the spectrum of host cell pathways that may be linked to the validated gene hits, pathway analysis was performed using Ingenuity Pathway Analysis software (Ingenuity Systems, http://www.ingenuity.com). The results provided network modeling of the validated genes and identified several functional groups (NF-κB, CRE/CREB signaling, apoptosis) linked to protease gene expression, host cell miRNA regulation, and influenza replication.

### Reporter system assays

Reporter plasmids used to confirm pathway were obtained from SABiosciences/Qiagen as a dual luciferase Cignal Reporter Assay Kit. The reporters consisted of a transcription factor of interest linked to a firefly luciferase gene, a positive control plasmid (luciferase linked to a CMV promoter), and a negative control plasmid (luciferase with no promoter). A549 cells were co-transfected with 100 nM of siRNA and 100 ng of the appropriate plasmid (reporter, positive, or negative control plasmids) using either the SureFECT (SABiosciences) or Attractene (Qiagen) transfection reagents. As a transfection control, the plasmid kit also contains a Renilla luciferase plasmid fused to a CMV promoter, and all data was normalized to Renilla luciferase expression. After 24 h of transfection, cells were allowed to rest for one day and then infected with A/WSN/33 (MOI = 0.001) for 24 h. Cell lysate was taken and luciferase expression was measured using a Safire2 Microplate reader (Tecan, Männedorf, Switzerland).

### Apoptosis array

A human apoptosis array was obtained from SABiosciences/Qiagen. A549 cells were transfected with 50 nM of either the appropriate siRNA or siNEG. After 48 h, cells were infected with A/WSN/33 at an MOI of 0.001. RNA was isolated 18 hpi, a time point chosen because it is late enough for apoptosis modulation to become evident at the transcriptional level, but sufficiently early such that cells were not destroyed [76], and then treated with DNase I to remove any genomic DNA. cDNA was synthesized using an RT^2^ First Strand Kit (SABiosciences) and PCR was performed using RT^2^ SYBR Green Master Mix (SABiosciences) per the manufacturer protocol. Data was analyzed by calculating 2^(−ΔΔCt)^. Silencing of the protease genes relative to siNEG-treated cells was confirmed for each independent experiment.

### miRNA studies

A library of miRNA hairpin inhibitors synthesized as RNA oligonucleotides with novel secondary structure designed to inhibit the function of endogenous miRNA, and chemically enhanced to improve efficacy and longevity (miRIDIAN, Dharmacon, Lafayette, CO) were used to target host cell miRNAs identified as potential regulators of host genes validated as important for influenza virus replication. miRNA mimics were not used since treatment with mimics results in extremely high concentrations of miRNA relative to biological levels. In these studies, A549 cells (1.5×10^4^) were transfected with 25 nM of an appropriate miRNA inhibitor using Dharmafect-1 per the manufacturer's protocol. After 48 h, the cells were infected with A/WSN/33 at an MOI of 0.001 for an additional 24 h or 48 h. Viral replication was assayed by TCID_50_ and qPCR for M gene as described above. Gene specific primers were also used to quantify changes in host gene expression in cells receiving the miRNA inhibitors using SybrGreen as described above.

### Statistics

The primary siRNA protease gene screen was performed independently and in duplicate. HA results for each gene were assigned values 0–8 based on the dilution of the HA readout. HA values were normalized to the average of the siNEG control readout per plate. Robust z-scores (*Z* = *xi−x/sx*) were used as the normalizing method and calculated for each gene to determine hits based upon the standard deviation where *xi* is the raw measurement on the *i*th siRNA, and *sx* are the mean and the standard deviation, respectively, of all measurements within the plate. Normalization of raw data removes systematic plate-to-plate variation, making measurements comparable across plates. Statistical analyses for the cross-strain validation studies, the reporter studies, and miRNA studies were performed using GraphPad Prism software using the Mann-Whitney U test.

## Supporting Information

Figure S1 **Calculated standard deviation of the z-scores of the human protease library.** The primary screen provided two independent studies and was analyzed using a scaling methodology that sets the non-targeting control siRNA (siNEG) at an arbitrary value of 1.0, and the negative control siTOX at zero. siRNAs targeting host genes were assigned a score based on the distribution of these values. Wells in the primary screen with a percent of differentiation greater than 1.5 standard deviations above the plate mean in both duplicates were considered primary hits. Of the 481 HP genes targeted, 24 were genes were identified as “primary hits”.
(TIF)

Figure S2 **siRNA treatment decreases protein levels of the target protease genes.** A: A549 cells were untreated (A549), treated with 50 nM of siNEG, or 50 nM of the appropriate siRNA. After 72 hours, cells were lysed in 1% TritonX-100 and protease gene levels were assessed by Western blot. B: Protein levels of the protease genes were determined by densitometry. Band density is shown as a percentage of the untreated A549 cells.
(TIF)

Figure S3 **An Ingenuity pathway analysis linked global cellular pathways and the host protease genes of interest.** Hit protease genes (ADAMTS7, CPE, DPP3, MST1, and PRSS12) are shown in red and relevant influenza proteins are shown in green. Nodes indicating global cellular pathways linked with the hit genes are shown in yellow (CREB, NF-κB, caspases).
(TIF)

Figure S4 **miRNAs interact with host protease genes.** An Ingenuity pathway analysis implicated several miRNAs connected with host protease genes of interest. Hit protease genes are shown in red and miRNAs are shown in green.
(TIF)

Table S1 **Expression ratios for pro- and anti-apoptotic genes in A549 cells treated with siRNA for protease gene targets^a,b^.** ^a^Gene expression in A549 cells treated with 50 nM of the appropriate siRNA was compared to cells treated with 50 nM of siNEG. The experiment was performed as described in Figure 4. ^b^Gene expression was normalized to GAPDH. *p<0.05.
(DOCX)
