# Supplementary figures and images for: MicroRNA Regulation of Human Protease Genes Essential for Influenza Virus Replication
Source: PLoS One. 2012 May 14;7(5):e37169. doi: 10.1371/journal.pone.0037169 (PMC3351457; doi:10.1371/journal.pone.0037169)

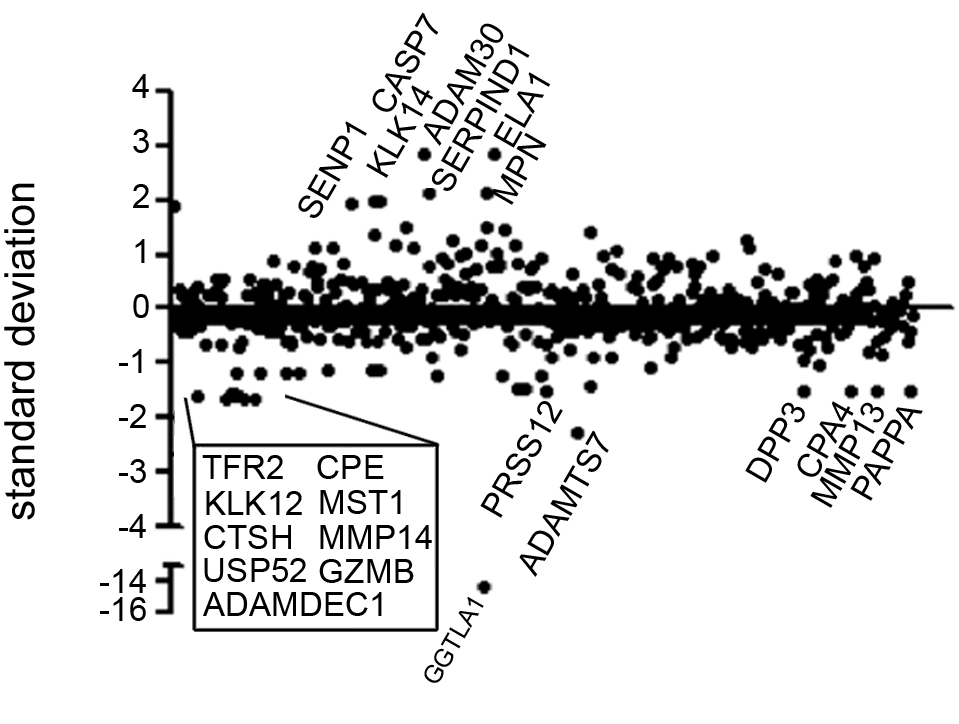

Supplement: Figure S1 — Calculated standard deviation of the z-scores of the human protease library. The primary screen provided two independent studies and was analyzed using a scaling methodology that sets the non-targeting control siRNA (siNEG) at an arbitrary value of 1.0, and the negative control siTOX at zero. siRNAs targeting host genes were assigned a score based on the distribution of these values. Wells in the primary screen with a percent of differentiation greater than 1.5 standard deviations above the plate mean in both duplicates were considered primary hits. Of the 481 HP genes targeted, 24 were genes were identified as “primary hits”. (TIF) [file pone.0037169.s001.tif]

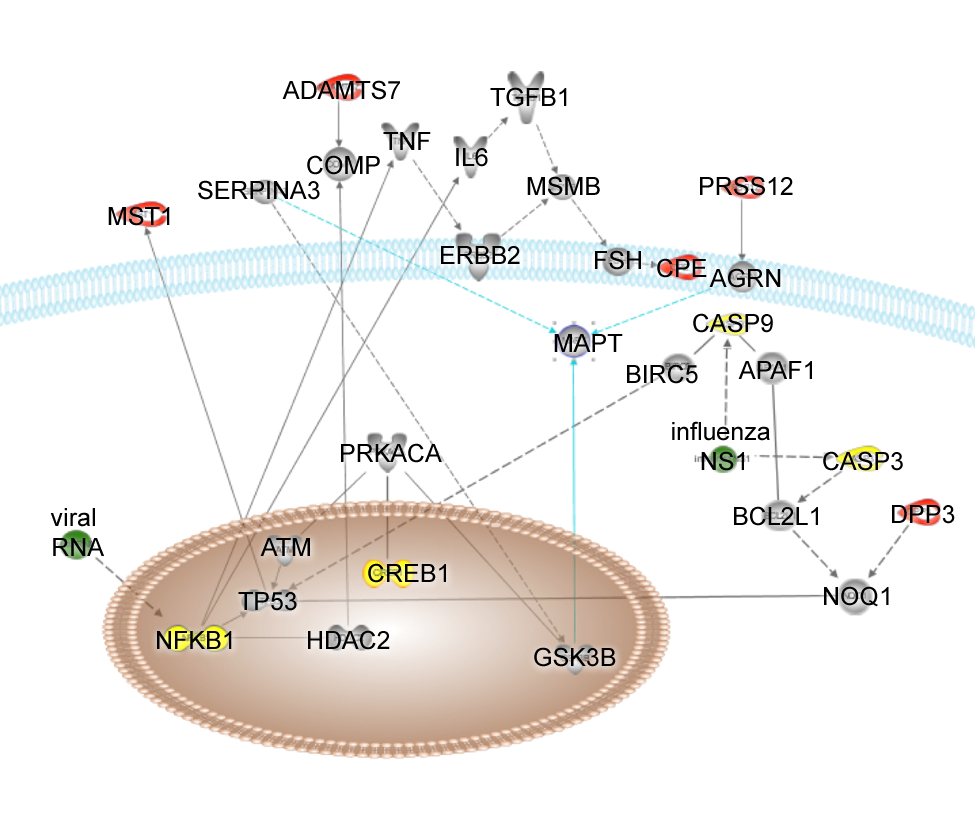

Supplement: Figure S3 — An Ingenuity pathway analysis linked global cellular pathways and the host protease genes of interest. Hit protease genes (ADAMTS7, CPE, DPP3, MST1, and PRSS12) are shown in red and relevant influenza proteins are shown in green. Nodes indicating global cellular pathways linked with the hit genes are shown in yellow (CREB, NF-κB, caspases). (TIF) [file pone.0037169.s003.tif]

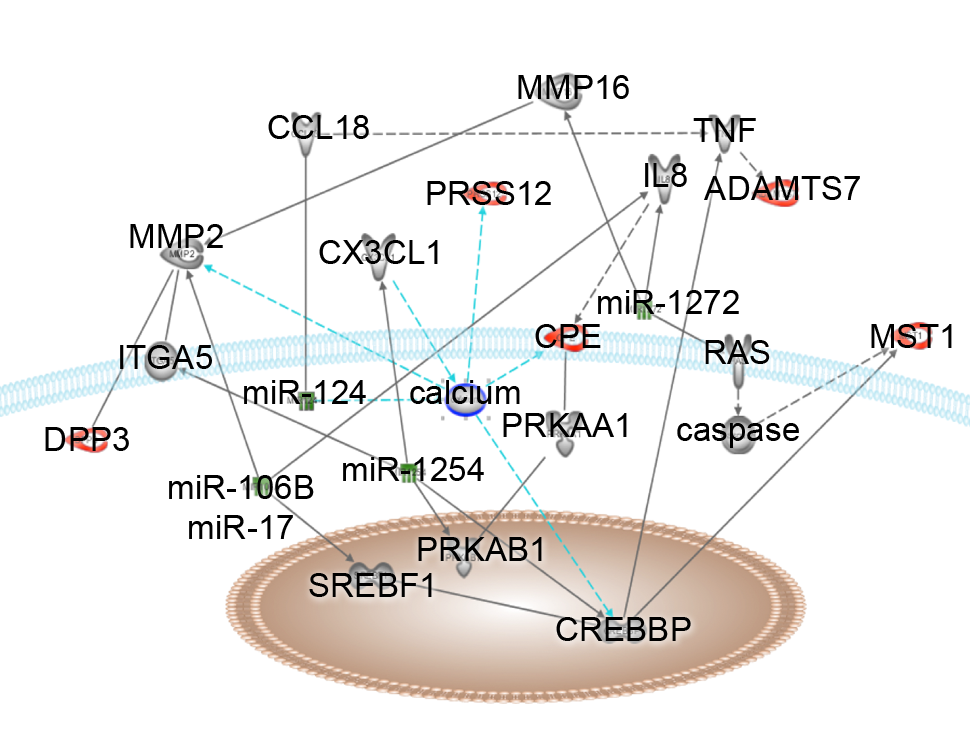

Supplement: Figure S4 — miRNAs interact with host protease genes. An Ingenuity pathway analysis implicated several miRNAs connected with host protease genes of interest. Hit protease genes are shown in red and miRNAs are shown in green. (TIF) [file pone.0037169.s004.tif]
