# Supplementary material for: MicroRNA Regulation of Human Protease Genes Essential for Influenza Virus Replication
Source: PLoS One. 2012 May 14;7(5):e37169. doi: 10.1371/journal.pone.0037169 (PMC3351457; doi:10.1371/journal.pone.0037169)
Supplement: Table S1 — Expression ratios for pro- and anti-apoptotic genes in A549 cells treated with siRNA for protease gene targetsa,b. aGene expression in A549 cells treated with 50 nM of the appropriate siRNA was compared to cells treated with 50 nM of siNEG. The experiment was performed as described in Figure 4. bGene expression was normalized to GAPDH. *p<0.05. (DOCX) [file pone.0037169.s005.docx]

Table S1: Expression ratios for pro- and anti-apoptotic genes in A549 cells treated with siRNA for protease gene targets^a,b^

| **Gene** | **siADAMTS7** | **siCPE** | **siDPP3** | **siMST1** | **siPRSS12** |
| --- | --- | --- | --- | --- | --- |
| ABL1 | 0.74 ± 0.02 | 0.69 ± 0.05 | 1.48 ± 0.36 | 0.73 ± 0.05 | 0.92 ± 0.09 |
| AKT1 | 0.95 ± 0.08 | 0.83 ± 0.08 | 1.12 ± 0.48 | 0.86 ± 0.23 | 1.28 ± 0.10 |
| APAF1 | 1.29 ± 0.05 | 1.07 ± 0.06 | 1.26 ± 0.42 | 1.20 ± 0.08 | 1.19 ± 0.09 |
| BAD | 1.04 ± 0.11 | 0.86 ± 0.03 | 1.43 ± 0.25 | 0.94 ± 0.12 | 1.07 ± 0.10 |
| BAG1 | 0.73 ± 0.02 | 0.46 ± 0.01 | 0.86 ± 0.19 | 0.59 ± 0.03 | 0.89 ± 0.15 |
| BAG3 | 0.80 ± 0.02 | 0.72 ± 0.07 | 0.91 ± 0.17 | 0.84 ± 0.07 | 0.73 ± 0.08 |
| BAG4 | 0.90 ± 0.07 | 1.05 ± 0.01 | 1.47 ± 0.68 | 1.27 ± 0.11 | 1.30 ± 0.12 |
| BAK1 | 0.49 ± 0.06 | 0.64 ± 0.06 | 0.78 ± 0.19 | 0.50 ± 0.09 | 0.63 ± 0.05 |
| BAX | 0.27 ± 0.01 | 0.28 ± 0.02 | 0.46 ± 0.19 | 0.45 ± 0.06 | 0.49 ± 0.05 |
| BCL10 | 1.10 ± 0.03 | 0.52 ± 0.05 | 0.95 ± 0.15 | 1.01 ± 0.02 | 0.76 ± 0.12 |
| BCL2 | 1.10 ± 0.12 | 1.09 ± 0.10 | 1.11 ± 0.40 | 1.24 ± 0.11 | 1.41 ± 0.12 |
| BCL2A1 | 0.50 ± 0.07 | 0.25 ± 0.02 | 1.15 ± 0.41 | 0.74 ± 0.04 | 0.33 ± 0.03 |
| BCL2L1 | 0.75 ± 0.08 | 0.50 ± 0.05 | 1.02 ± 0.54 | 0.77 ± 0.18 | 0.90 ± 0.15 |
| BCL2L10 | 0.87 ± 0.07 | 0.65 ± 0.07 | 5.90 ± 3.79 | 1.15 ± 0.28 | 1.23 ± 0.23 |
| BCL2L11 | 0.81 ± 0.03 | 0.55 ± 0.04 | 1.22 ± 0.16 | 0.56 ± 0.04 | 0.64 ± 0.10 |
| BCL2L2 | 0.98 ± 0.05 | 0.92 ± 0.01 | 0.79 ± 0.14 | 0.93 ± 0.06 | 1.11 ± 0.10 |
| BCLAF1 | 0.72 ± 0.06 | 0.75 ± 0.01 | 0.98 ± 0.18 | 0.76 ± 0.04 | 0.84 ± 0.07 |
| BFAR | 1.13 ± 0.07 | 1.03 ± 0.03 | 1.33 ± 0.34 | 1.07 ± 0.08 | 1.11 ± 0.07 |
| BID | 0.87 ± 0.04 | 0.78 ± 0.03 | 1.30 ± 0.38 | 0.82 ± 0.04 | 0.97 ± 0.13 |
| BIK | 0.26 ± 0.01 | 0.27 ± 0.02 | 0.48 ± 0.13 | 0.28 ± 0.07 | 0.31 ± 0.05 |
| NAIP | 0.71 ± 0.09 | 0.68 ± 0.06 | 0.64 ± 0.06 | 1.26 ± 0.07 | 0.95 ± 0.12 |
| BIRC2 | 1.17 ± 0.03 | 0.82 ± 0.06 | 0.78 ± 0.11 | 0.93 ± 0.04 | 0.90 ± 0.08 |
| BIRC3 | 0.67 ± 0.05 | 0.95 ± 0.12 | 0.96 ± 0.23 | 1.06 ± 0.01 | 0.81 ± 0.07 |
| BIRC4 | 0.77 ± 0.10 | 0.63 ± 0.11 | 0.93 ± 0.17 | 0.72 ± 0.08 | 0.88 ± 0.09 |
| BIRC6 | 0.69 ± 0.02 | 0.47 ± 0.07 | 0.90 ± 0.08 | 0.61 ± 0.02 | 0.53 ± 0.08 |
| BIRC8 | 0.93 ± 0.12 | 0.74 ± 0.08 | 3.01 ± 1.44 | 1.31 ± 0.32 | 1.40 ± 0.26 |
| BNIP1 | 0.81 ± 0.03 | 0.57 ± 0.02 | 0.82 ± 0.11 | 0.53 ± 0.01 | 0.57 ± 0.02 |
| BNIP2 | 1.00 ± 0.05 | 0.73 ± 0.01 | 0.95 ± 0.26 | 1.05 ± 0.08 | 0.97 ± 0.13 |
| BNIP3 | 0.99 ± 0.06 | 0.87 ± 0.02 | 0.88 ± 0.09 | 0.92 ± 0.001 | 1.01 ± 0.08 |
| BNIP3L | 1.61 ± 0.03 | 1.36 ± 0.07 | 1.97 ± 0.31 | 1.65 ± 0.08 | 1.64 ± 0.14 |
| BRAF | 0.77 ± 0.05 | 0.65 ± 0.03 | 0.82 ± 0.10 | 0.89 ± 0.01 | 0.69 ± 0.05 |
| NOD1 | 1.10 ± 0.09 | 0.65 ± 0.06 | 1.16 ± 0.20 | 0.85 ± 0.14 | 0.79 ± 0.07 |
| CARD6 | 1.00 ± 0.02 | 0.54 ± 0.01 | 1.20 ± 0.19 | 0.45 ± 0.05 | 0.44 ± 0.10 |
| CARD8 | 0.56 ± 0.11 | 0.51 ± 0.10 | 0.79 ± 0.16 | 0.51 ± 0.11 | 0.69 ± 0.11 |
| CASP1 | 1.13 ± 0.41 | 0.45 ± 0.12 | 2.20 ± 0.49 | 0.69 ± 0.08 | 0.58 ± 0.14 |
| CASP10 | 0.91 ± 0.07 | 0.61 ± 0.06 | 0.93 ± 0.19 | 0.75 ± 0.08 | 1.10 ± 0.19 |
| CASP14 | 0.93 ± 0.12 | 0.74 ± 0.08 | 1.42 ± 0.98 | 1.31 ± 0.32 | 1.40 ± 0.26 |
| CASP2 | 1.20 ± 0.05 | 0.86 ± 0.05 | 1.12 ± 0.37 | 1.09 ± 0.02 | 1.26 ± 0.15 |
| CASP3 | 0.55 ± 0.05 | 0.39 ± 0.001 | 0.83 ± 0.10 | 0.55 ± 0.03 | 0.48 ± 0.04 |
| CASP4 | 1.62 ± 0.06 | 0.55 ± 0.05 | 1.71 ± 0.34 | 0.71 ± 0.05 | 0.97 ± 0.20 |
| CASP5 | 0.60 ± 0.22 | 0.54 ± 0.26 | 1.20 ± 0.59 | 0.89 ± 0.74 | 1.40 ± 0.26 |
| CASP6 | 0.88 ± 0.06 | 0.60 ± 0.01 | 1.00 ± 0.24 | 0.66 ± 0.03 | 0.60 ± 0.05 |
| CASP7 | 1.82 ± 0.09 | 0.88 ± 0.05 | 2.04 ± 0.77 | 0.72 ± 0.04 | 0.92 ± 0.11 |
| CASP8 | 1.12 ± 0.14 | 0.88 ± 0.06 | 1.80 ± 0.81 | 0.90 ± 0.04 | 0.83 ± 0.05 |
| CASP9 | 1.19 ± 0.16 | 0.65 ± 0.03 | 1.25 ± 0.04 | 0.84 ± 0.03 | 1.22 ± 0.17 |
| CD40 | 1.22 ± 0.14 | 0.83 ± 0.03 | 1.74 ± 0.50 | 1.11 ± 0.09 | 1.45 ± 0.19 |
| CD40LG | 0.93 ± 0.12 | 0.74 ± 0.08 | 1.42 ± 0.98 | 1.31 ± 0.32 | 1.40 ± 0.26 |
| CFLAR | 1.00 ± 0.07 | 0.61 ± 0.06 | 1.37 ± 0.43 | 1.06 ± 0.06 | 0.93 ± 0.10 |
| CIDEA | 0.93 ± 0.12 | 0.74 ± 0.08 | 0.35 ± 0.18 | 0.71 ± 0.29 | 1.40 ± 0.26 |
| CIDEB | 1.33 ± 0.18 | 0.90 ± 0.02 | 1.19 ± 0.38 | 1.05 ± 0.08 | 1.10 ± 0.20 |
| CRADD | 0.99 ± 0.07 | 0.73 ± 0.04 | 0.73 ± 0.10 | 0.52 ± 0.03 | 0.80 ± 0.10 |
| DAPK1 | 0.68 ± 0.03 | 0.56 ± 0.02 | 0.94 ± 0.21 | 0.59 ± 0.01 | 0.66 ± 0.08 |
| DFFA | 0.70 ± 0.03 | 0.56 ± 0.02 | 0.59 ± 0.08 | 0.64 ± 0.02 | 0.63 ± 0.04 |
| FADD | 1.19 ± 0.08 | 1.26 ± 0.07 | 1.24 ± 0.42 | 1.20 ± 0.21 | 1.32 ± 0.05 |
| FAS | 0.72 ± 0.05 | 0.45 ± 0.04 | 0.96 ± 0.37 | 0.69 ± 0.05 | 0.66 ± 0.07 |
| FASLG | 0.93 ± 0.12 | 0.74 ± 0.08 | 1.42 ± 0.98 | 1.31 ± 0.32 | 0.71 ± 0.59 |
| GADD45A | 0.64 ± 0.02 | 0.27 ± 0.02 | 0.77 ± 0.19 | 0.54 ± 0.001 | 0.60 ± 0.07 |
| HRK | 0.67 ± 0.09 | 0.47 ± 0.08 | 0.40 ± 0.07 | 0.64 ± 0.02 | 1.16 ± 0.12 |
| IGF1R | 0.75 ± 0.04 | 0.72 ± 0.06 | 1.04 ± 0.17 | 0.52 ± 0.01 | 0.71 ± 0.10 |
| LTA | 0.92 ± 0.12 | 0.73 ± 0.08 | 0.52 ± 0.34 | 1.29 ± 0.31 | 1.02 ± 0.49 |
| LTBR | 1.11 ± 0.15 | 0.65 ± 0.03 | 0.98 ± 0.24 | 0.65 ± 0.001 | 0.93 ± 0.08 |
| MCL1 | 0.97 ± 0.04 | 0.98 ± 0.05 | 1.49 ± 0.31 | 1.02 ± 0.05 | 0.88 ± 0.08 |
| NOL3 | 1.22 ± 0.10 | 1.18 ± 0.10 | 1.74 ± 0.42 | 1.17 ± 0.08 | 1.75 ± 0.07 |
| PYCARD | 0.60 ± 0.03 | 0.39 ± 0.02 | 1.00 ± 0.11 | 0.59 ± 0.01 | 0.58 ± 0.04 |
| RIPK2 | 1.15 ± 0.04 | 0.64 ± 0.02 | 0.90 ± 0.16 | 0.69 ± 0.01 | 0.85 ± 0.09 |
| TNF | 0.56 ± 0.07 | 0.45 ± 0.05 | 1.65 ± 0.78 | 0.63 ± 0.35 | 0.89 ± 0.29 |
| TNFRSF10A | 1.03 ± 0.04 | 0.58 ± 0.02 | 0.80 ± 0.23 | 0.70 ± 0.01 | 0.75 ± 0.12 |
| TNFRSF10B | 1.13 ± 0.07 | 0.87 ± 0.02 | 0.89 ± 0.26 | 0.94 ± 0.02 | 0.83 ± 0.06 |
| TNFRSF11B | 2.27 ± 0.25 | 2.35 ± 0.28 | 1.19 ± 0.34 | 0.97 ± 0.10 | 2.33 ± 0.33 |
| TNFRSF1A | 1.14 ± 0.09 | 0.73 ± 0.04 | 1.58 ± 0.34 | 0.93 ± 0.01 | 1.00 ± 0.13 |
| TNFRSF21 | 2.11 ± 0.11 | 0.90 ± 0.02 | 1.04 ± 0.32 | 0.98 ± 0.01 | 0.92 ± 0.09 |
| TNFRSF25 | 1.24 ± 0.37 | 0.60 ± 0.13 | 6.40 ± 3.02 | 1.05 ± 0.27 | 1.04 ± 0.21 |
| CD27 | 0.59 ± 0.10 | 0.38 ± 0.05 | 2.30 ± 1.22 | 0.60 ± 0.27 | 0.89 ± 0.53 |
| TNFRSF9 | 0.88 ± 0.08 | 1.20 ± 0.06 | 1.70 ± 0.66 | 1.03 ± 0.10 | 0.93 ± 0.31 |
| TNFSF10 | 7.50 ± 2.11 | 1.12 ± 0.34 | 6.32 ± 1.33* | 0.75 ± 0.04 | 0.94 ± 0.35 |
| CD70 | 1.47 ± 0.22 | 0.36 ± 0.001 | 0.90 ± 0.05 | 0.57 ± 0.01 | 0.88 ± 0.28 |
| TNFSF8 | 1.86 ± 0.39 | 0.68 ± 0.13 | 20.77 ± 5.79* | 1.31 ± 0.32 | 1.19 ± 0.36 |
| TP53 | 0.59 ± 0.05 | 0.75 ± 0.02 | 0.89 ± 0.23 | 0.77 ± 0.05 | 1.03 ± 0.14 |
| TP53BP2 | 0.62 ± 0.04 | 0.61 ± 0.001 | 0.71 ± 0.16 | 0.65 ± 0.07 | 0.80 ± 0.19 |
| TP73 | 0.76 ± 0.22 | 0.74 ± 0.08 | 0.32 ± 0.10 | 1.31 ± 0.32 | 1.01 ± 0.50 |
| TRADD | 0.93 ± 0.04 | 0.53 ± 0.02 | 1.19 ± 0.23 | 0.75 ± 0.05 | 0.96 ± 0.17 |
| TRAF2 | 0.58 ± 0.02 | 0.88 ± 0.04 | 1.50 ± 0.76 | 1.06 ± 0.20 | 1.19 ± 0.14 |
| TRAF3 | 0.83 ± 0.04 | 0.83 ± 0.03 | 0.96 ± 0.33 | 1.11 ± 0.06 | 1.21 ± 0.23 |
| TRAF4 | 0.73 ± 0.04 | 0.60 ± 0.01 | 0.71 ± 0.31 | 0.65 ± 0.06 | 1.02 ± 0.26 |

^a^Gene expression in A549 cells treated with 50 nM of the appropriate siRNA was compared to cells treated with 50 nM of siNEG. The experiment was performed as described in Figure 4.

^b^Gene expression was normalized to GAPDH.

*p < 0.05
